# Glia: the cellular glue that binds circadian rhythms and sleep

**DOI:** 10.1093/sleep/zsae314

**Published:** 2025-01-15

**Authors:** Catarina Carvalhas-Almeida, Amita Sehgal

**Affiliations:** CNC-UC—Center for Neuroscience and Cell Biology, University of Coimbra, Coimbra, Portugal; CIBB—Centre for Innovative Biomedicine and Biotechnology, University of Coimbra, Coimbra, Portugal; Faculty of Pharmacy, University of Coimbra, Coimbra, Portugal; Chronobiology and Sleep Institute, Perelman School of Medicine at the University of Pennsylvania, Philadelphia, PA, USA; Chronobiology and Sleep Institute, Perelman School of Medicine at the University of Pennsylvania, Philadelphia, PA, USA; Howard Hughes Medical Institute, University of Pennsylvania, Philadelphia, PA, USA

**Keywords:** glia cells, sleep, circadian rhythms, mammals, *Drosophila*

## Abstract

Glia are increasingly appreciated as serving an important function in the control of sleep and circadian rhythms. Glial cells in *Drosophila* and mammals regulate daily rhythms of locomotor activity and sleep as well as homeostatic rebound following sleep deprivation. In addition, they contribute to proposed functions of sleep, with different functions mapping to varied glial subtypes. Here, we discuss recent findings in *Drosophila* and rodent models establishing a role of glia in circadian or sleep regulation of synaptic plasticity, brain metabolism, removal of cellular debris, and immune challenges. These findings underscore the relevance of glia for benefits attributed to sleep and have implications for understanding the neurobiological mechanisms underlying sleep and associated disorders.

## Statement of Significance

This review addresses the role of glial cells in regulating sleep and circadian rhythms, detailing their contributions to synaptic plasticity, metabolism, and immune responses. By providing a timeline of key discoveries from the past two decades, it highlights the progressive understanding of glial functions in sleep regulation. Insights have come from *Drosophila* and rodent models, emphasizing the evolutionary importance of glia in restorative functions of sleep. These findings deepen our understanding of sleep biology and have significant implications for addressing sleep-related disorders.

## Historical Overview

In both mammals and *Drosophila*, glial cells form a diverse class of non-neuronal cells crucial for nervous system function. The term “glia,” derived from the Greek word for “glue,” alludes to their structural support role [1]. Mammalian glial classes include astrocytes, oligodendrocytes, microglia, and Schwann cells, each fulfilling distinct roles in supporting neuronal structure and function, regulating the extracellular environment, and contributing to immune responses [2]. In *Drosophila*, glial classes such as astrocyte-like glia, wrapping glia, surface glia, and cortex glia serve analogous functions with some degree of variation [3]. Despite species-specific differences, these cells promote neuronal health, facilitate signal transmission, modulate synaptic plasticity, regulate neuroinflammatory responses, and maintain metabolic and neural homeostasis [4–7].

Sleep, a behavior conserved between species, is regulated by homeostatic mechanisms and the circadian clock [8, 9]. Sleep homeostasis controls sleep needs, the amount and intensity of which are proportional to prior wake time [10]. The daily timing of sleep is driven by circadian clocks, which are molecular oscillators comprised of transcriptional and translational feedback loops [11]. The activity of clock genes within these loops times the expression of a multitude of genes and processes in a 24-hour cycle [12–14], thereby allocating specific functions to specific times of the day [11].

Although early studies from the 1990s first identified clock gene expression in glia [15], it wasn’t till much later that key functions of the clock in different glial subtypes were identified. For instance, clocks in mammalian astrocytes were shown to sustain circadian behavioral rhythms in the absence of neurons, and circadian changes in microglial morphology were implicated in brain surveillance, regulation of neuroinflammation, and phagocytosis of damaged neurites [16–20].

Indeed, over the past two decades, significant advances have been made in understanding the influence of glial function on sleep–wake cycles, as well as sleep depth and duration [21–23], and other circadian rhythms. In the early 2000s, astrocytes were shown to exhibit circadian rhythms [24], with studies demonstrating that glutamate uptake [25, 26] and extracellular adenosine triphosphate (ATP) release by astrocytes are regulated by clock genes [27]. A γ-aminobutyric acid (GABA) metabolic enzyme in *Drosophila* glia was shown to modulate sleep in *sleepless* mutants [28]. Astrocytic exocytosis was found to impact mammalian sleep regulation [29] and microglia were shown to express clock genes that regulate synaptic plasticity and morphology [30, 31]. Proliferation and differentiation of oligodendrocyte precursor cells were found to vary with sleep and activity in different regions of the mouse brain [32, 33]. Furthermore, the loss of a clock gene in astrocytes was linked to neuroinflammation and, conversely, inflammatory signaling in astrocytes was associated with changes in sleep [34, 35]. In 2016, adenosine kinase in astrocytes was identified as a regulator of sleep homeostasis [36].

We now know that glial regulation of synaptic plasticity, GABA homeostasis, and metabolic regulation is influenced by sleep–wake cycles [37–43]. In 2018, studies in *Drosophila* revealed sleep-modulated endocytosis in glia of the blood–brain barrier (BBB) [44] and circadian regulation of xenobiotic efflux in the BBB, which is also conserved in mice [45, 46]. The regulation of circadian behavior by astrocyte microRNAs was also described [47].

More recently, in 2022, it was demonstrated that glial cells control sphingolipid dynamics for circadian circuit remodeling [48] and that *BMAL1* is essential for astrocyte endolysosomal function [49]. In 2023, lipid and carnitine transporters in glia were found to regulate sleep in *Drosophila* [50], and ecdysone was found to modulate sleep via lipid metabolism in cortex glia [51]. In the current year, microglia were shown to regulate sleep through Ca^2+^-dependent interactions [52], sleep-dependent lipid transfer from neurons to glia was found to be vital for mitochondrial health [53] and transcriptomic studies suggested that glial cells integrate circadian and homeostatic regulation of sleep [54].

**Figure 1.**
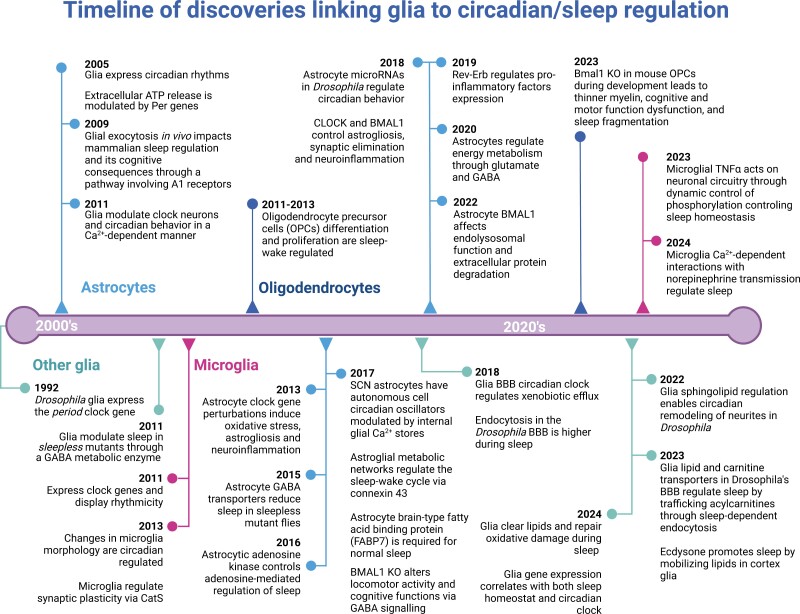
Timeline of key findings on glial sleep and circadian rhythms from rodent and *Drosophila* models since 1990.

All these studies and others described below are summarized in a timeline in Figure 1. As earlier studies were reviewed comprehensively by Artiushin et al. [55], we focus here largely on discoveries since 2020, highlighting the most recent advances in the role of glia in mammalian and *Drosophila* circadian rhythms and sleep.

## Regulation of Circadian and Homeostatic Sleep Behavior by Glia

### Astrocytes in *Drosophila* and mammals

In mammals and flies, circadian regulation by astrocytes is mediated, at least in part, through Ca^2+^ signaling [16, 18]. Studies in both models show that Ca^2+^ fluctuations correlate with sleep need, and are necessary for sleep rebound [56–58]. In *Drosophila*, Ca^2+^ fluctuations are regulated through a positive feedback loop involving the monoamine receptor TyrRII, a G-coupled receptor (GPCR) [56]. Similarly in the mouse cortex, astrocytes control slow-wave oscillations through specific Ca^2+^ signaling GPCR pathways, with a Gi-GPCR controlling sleep depth and Gq-GPCR affecting the frequency of sleep-awake transitions [23]. In the basal forebrain, the Gq-GPCR pathway promotes prolonged wakefulness without triggering the typical rebound response to sleep loss. These observations suggest that the triggering of sleep needs by wakefulness requires the selective activation of distinct neuronal-glial circuits during wake [59]. The contrasting roles of different G-protein mediated pathways indicate the complexity of sleep-related Ca^2+^ dynamics in astrocytes across brain regions.

Moreover, distinct clusters of astrocytes may have unique sleep-related Ca^2+^ signaling properties, as tested using optical fiber photometry in freely behaving mice. Ca^2+^ oscillations across brain regions (cortex, hippocampus, hypothalamus, cerebellum, and pons) showed a universal decrease in Ca^2+^ activity during rapid eye movement (REM) sleep and an increase upon awakening, with non-REM (NREM)-linked activity varying across regions [60]. Prolonged low-amplitude astrocytic Ca^2+^ elevations during sleep are linked to extracellular K^+^ dynamics, while shorter, pronounced Ca^2+^ surges during wakefulness are noradrenergic-dependent and involve the IP_3_ pathway [61].

In mammals, astrocyte-neuron interactions within the hypothalamus are particularly critical for sleep and circadian regulation [62–64]. Specifically, in the ventrolateral preoptic nucleus (VLPO), astrocytes promote sleep by releasing adenosine, which activates GABAergic sleep-promoting neurons that reduce neuronal excitability in arousal-related brain regions [62, 64]. This process is facilitated by astrocytic-ATP, converted to adenosine to act on VLPO neurons to promote sleep [64]. In the lateral hypothalamus (LH), astrocytes exhibit increased activity during transitions from NREM sleep to wakefulness, as recorded during spontaneous sleep–wake cycles in freely moving mice. Additionally, chemogenetic activation of LH astrocytes promotes arousal, sustains prolonged wakefulness, and significantly elevates Ca^2+^ activity in adjacent GABAergic neurons, which are known to promote wakefulness [65, 66], suggesting that LH astrocytes play a key role in sleep-wake regulation by modulating these surrounding neurons [63]. Others have shown that astrocytic Ca^2+^ signals precede NREM sleep-to-wakefulness transitions in the cortex [58]. Cortical astrocyte activation likely precedes astrocytic activation in subcortical regions, such as the LH, during NREM sleep-to-wake transitions, suggesting that astrocytes in the central nervous system regulate arousal from “top-to-bottom” by acting on specific neocortical circuits, such as the ones involved in local sleep, and by regulating sleep/ arousal centers located in the basal forebrain and hypothalamus, contributing to global sleep [63, 67]. Local sleep occurs in anatomically discrete brain locations in vivo or in cultured neuronal networks [68, 69] and suggests that sleep can be initiated by small networks of neurons and glial cells [70, 71]. Local and global sleep are defined by electrophysiological and homeostatic measures such as delta power, synchronization of activity, action potential burstiness, and the tendency to sleep excessively after prolonged wakefulness [68]. Two-photon imaging of astrocytic responses across various spatiotemporal scales found that neurotransmitters such as GABA and glutamate trigger widespread, long-lasting Ca^2+^ responses in astrocytes, extending beyond the initially stimulated cell, thus, influencing subsequent neuronal activity [70]. This local-to-global concept may also apply to circadian synchronization. Complementary findings using microfluidic devices identified astrocytes as active participants in synchronizing molecular clocks between distant neuronal populations through GABA and glutamate signaling [71]. Thus, local astrocytic networks, which encode transient neurotransmitter inputs, play a pivotal role in maintaining local network synchrony, potentially leading to long-range signal transmission through yet unidentified mechanisms [70, 72].

Beyond Ca^2+^ signaling, astrocytes contribute to sleep and circadian regulation through various factors. For instance, in flies, the *Drosophila melanogaster* mesencephalic astrocyte-derived neurotrophic factor (DmMANF), implicated in circadian regulation, is expressed in ensheathing glia and astrocytes, impacting sleep and activity patterns by influencing the projections of pigment dispersing factor (PDF)-expressing clock neurons [73]. Reducing DmMANF expression in glia disrupts the locomotor activity rhythm, alters the morphology of dorsal projections of the small PDF cells, and reduces arborizations of large PDF cells in the optic lobe medulla. Additionally, DmMANF shapes the morphology of the L2 interneurons in the visual system, regulating the circadian rhythm of their dendritic tree topology, underscoring its importance in neurite development and circadian plasticity [74].

The enzyme arylalkylamine *N*-acetyltransferase 1 (AANAT1), which inactivates monoamines by acetylating them, regulates serotonin and dopamine accumulation in the brain during sleep deprivation [75]. Depleting AANAT1 specifically from astrocytes, but not neurons, increased daytime recovery sleep after overnight sleep deprivation in flies, emphasizing its role in regulating monoamine availability and homeostatic sleep [76]. Additionally, the loss of the astrocytic GABA transporter (GAT) in a hypomorphic *gat33-1* mutant, reduced GABAergic tone, leading to prolonged sleep latency, and disrupted sleep homeostasis. Combining the mutation of *gat33-1* with mutations in genes that regulate cell-surface levels of GABA_A_ receptor resistance to dieldrin (RDL) in the wake-promoting large ventral lateral neurons (l-LNvs) suppressed the *gat33-1* phenotype [77]. Altogether, multiple factors and pathways orchestrate the molecular mechanisms of circadian rhythm and homeostatic sleep regulation by astrocytes.

### Other glial cell types in *Drosophila* and mammals

The literature on microglia and other glial cells in sleep regulation is still limited, and their roles are just beginning to be understood. The Gi-GPCR pathway in microglia appears to be important for sleep, with chemogenetic activation in mice promoting sleep by increasing microglial intracellular Ca^2+^ levels and pharmacological blockade of Gi-coupled P2Y12 receptors decreasing Ca^2+^ and sleep [52]. Increased microglial Ca^2+^ levels during natural wake-to-sleep transitions are thought to result from reduced norepinephrine levels and increased adenosine concentration, indicating that microglia regulate sleep through interactions with norepinephrine transmission [52].

Microglia also appear to regulate wakefulness, perhaps as a result of their effects on sleep [78–81]. Depletion of microglia has been shown to reduce stable nighttime wakefulness by increasing wakefulness-NREM sleep transitions [78]. Others have also shown that under baseline conditions, microglial depletion reduced the wake amount and intensity, which are fully reversed after microglia repopulation. Additionally, under psychosocial stress conditions promoted by acute social defeat stress, microglia depletion promoted stress-induced sleep rebound, and exacerbated vulnerability to acute psychosocial stress [79], indicating that microglia may actively modulate sleep/wake cycles under both baseline and stress conditions. This effect seems to be mediated through the modulation of ceramide levels, which are normally lower during wakefulness than sleep in control mice. In the absence of microglia, this rhythmicity is disrupted, so it is possible that microglia modulate diurnal ceramide fluctuations in the brain to maintain stable wakefulness at night. Specifically, microglia in the thalamic reticular nucleus (TRN) appear to influence stable wakefulness via ceramide signaling to anterior TRN neurons, and chemogenetic activation of these neurons restores wakefulness stability affected by microglial depletion [78]. Microglia also regulate homeostatic rebound following sleep deprivation, at least in part by releasing tumour necrosis factor alpha (TNFα), which mediates protein phosphorylation in the cortex. The deletion of microglial TNFα disrupts this phosphorylation, impairing sleep rebound [82].

The presence of an intrinsic clock in microglial cells is well-documented, but its influence on the neuronal clock machinery and central nervous system (CNS) rhythms is debatable. Conflicting evidence is reported with some studies showing that acute depletion of brain microglia in adult rats disrupts diurnal rhythms of temperature, motor activity, and metabolism and dysregulates the clock machinery in the suprachiasmatic nucleus (SCN) and the hippocampus [83], whereas other studies demonstrate that clock gene expression in the cortex remains intact following microglia depletion in mice [84] and neither the free-running period under constant darkness nor light entrainment under jet-lag conditions are affected [85]. Moreover, an in vitro study showed that microglia depletion significantly reduces the amplitude of the SCN clock, whereas co-culturing SCN explants with resting microglia polarized to an anti-inflammatory phenotype (M2) improved the amplitude of the SCN clock [86], suggesting that M2-polarized microglia positively affect SCN clock function. The discrepancies between these studies may be due to differences in experimental conditions (in vivo vs. in vitro), species used (rats vs. mice), brain regions examined, or the methods of microglial depletion. More comprehensive and controlled studies are needed to clarify the exact role of microglia in circadian regulation.

Some of these roles of glia in circadian rhythm regulation are conserved. In *Drosophila*, behavioral rhythms are driven by the clock network in the brain, but glial cells contribute to circadian regulation. Astrocyte-like glia and chiasm giant glia, a specific glial cell type located in the optic lobe that helps carry visual information to the *Drosophila* eyes, are necessary to maintain daily variations in the arborizations of clock neurons, whereas epithelial glia, another specialized glial class in the fly’s visual system, regulate the amplitude of these changes [87, 88]. Oscillators in specific types of glia, including astrocyte-like glia, epithelial glia, and subperineural glia of the BBB, maintain wakefulness during the day by regulating the daily plasticity of clock neurons, thus controlling sleep amount. Additionally, the communication between glia and neurons through tripartite synapses involving epithelial glia is important for promoting wakefulness during the day [87].

Single-cell RNA sequencing in mice and *Drosophila* has shed light on the relationship between sleep and glial gene expression in the brain [54, 89]. In mice, different cell types such as astrocytes, neurons, endothelial cells, and microglia exhibit distinct transcriptional responses to sleep needs across brain regions [89]. In addition, astrocyte-specific RNA sequencing from mice across six time points revealed strong molecular rhythms within the nucleus accumbens, with 43.3% of the astrocyte transcriptome, including the canonical clock genes and several metabolic genes, showing significant diurnal rhythmicity [90]. Circadian gene expression in *Drosophila* glia suggests an integration of homeostatic and circadian processes within these cells, which could be critical to regulate sleep–wake cycles; indeed, disruptions in glial clock genes impaired sleep rebound after deprivation [54]. The idea that glia integrates homeostatic and circadian influences on sleep emphasizes the importance of studying these cells to address key outstanding questions in the circadian/sleep field.

## Glial Cells and Potential Functions of Sleep

### Synaptic plasticity

Synaptic plasticity refers to the capacity of synapses to undergo long-lasting changes in their efficacy and strength and is crucial for learning, memory, and neural adaptability [91]. Glial cells influence synaptic plasticity by providing metabolic support, facilitating glutamate uptake, clearing synaptic debris via exocytosis and phagocytosis [4, 40], and promoting non-Hebbian synaptic potentiation through increased concentrations of the glia-derived cytokine TNF-α [92]. Synaptic plasticity is also sleep–wake modulated with growing evidence adding glial cells to this regulation [93]. One of the best examples is provided by astrocytic and microglial regulation of ocular dominance plasticity, a type of visual cortical plasticity dependent on sleep and requiring both synaptic weakening and strengthening [14].

Interestingly, sleep deprivation promotes an accumulation of damaged neurites in both flies and mammals [20, 40]. In *Drosophila*, treatment with gaboxadol, a sleep-promoting drug, accelerates neurite clearance, while mechanical and genetic sleep deprivation impaired the clearance of damaged neurites. Genetically promoting sleep increased the expression of the phagocytosis marker *Draper* in ensheathing glia, providing a new mechanism for sleep-dependent modulation of glial function and neurite clearance [20]. The catabolization of sphingolipids to produce glucocerebrosidase in glial cells can also influence synaptic plasticity; indeed, both sphingolipid biosynthesis and degradation were required for the diurnal remodeling of circadian clock neurites, which grow and shrink across the day [48].

Synaptic plasticity is linked to neurogenesis, axon guidance, and synaptic signaling, with the Eph/ephrin tyrosine kinase receptor pathway playing a crucial role [94]. Eph/ephrin knocked down in neurons, but not in glia, disrupts mushroom body development in *Drosophila*. However, both neuronal and glial knockdowns alter sleep/circadian rhythms [95]. In a *Drosophila* model of Alzheimer’s disease (AD) based on the AD-associated *Eph*^*A1WT*^ and *Eph*^*A1P460L*^ mutations, manipulation of *Eph*/*ephrin* levels in neurons affects sleep, neurophysiology and, memory, potentially linking these effects to AD pathology [96]. Notably, *EPHA1* mutations in mammalian microglia are implicated in AD, suggesting a conserved role of *Eph/ephrin* signaling in cognitive function, at least partly through glia and perhaps via effects on sleep [97].

Recent studies in mammals have increasingly highlighted the role of microglia in sleep-dependent synaptic plasticity. For instance, microglial morphology and microglia-spine contacts are modulated by the sleep/wake cycle and synaptic activity through Cx3cr1 signaling [80, 98]. During wakefulness, surveillant microglia migrate towards active spines, while during sleep, predominantly during NREM sleep, they increase contact with synapses via their ramifications, suggesting that microglia shape their morphology and role based on neuronal activity and vigilance states [98]. Microglial morphology also shows regional differences with sleep–wake states, such that cortical microglial cell sizes negatively correlate with NREM sleep slow-wave activity; this is not true for the microglia found in the hippocampus and basal forebrain [99]. Furthermore, microglia depletion in mice extends NREM sleep, reduces hippocampal CA1 excitatory neurotransmission in the dark, and increases long-term potentiation during the light phase (ZT4) via CX3CR1. The expression levels of *cx3cr1* in microglia decrease during the light phase and in vitro upon exposure to ATP, a purinergic molecule released during sleep, suggesting a phase-dependent modulation of synaptic activity by microglia through *cx3cr1* regulation [80].

A proposed mechanism for eliminating weak synapses during sleep involves circadian changes in glucocorticoids and norepinephrine, affecting microglial activity and synaptic strength [100, 101]. At sleep onset in rats (ZT0), reduced synaptic proteins in the rat prefrontal cortex and activated microglia suggest microglial priming for phagocytosis. This is further emphasized by higher synapsin I protein levels in microglia, suggesting phagocytosis of presynaptic proteins [102]. At ZT0, isolated microglia contained approximately 25% more synapsin I protein than those at ZT12; however, both timepoints contained significant amounts of synapsin I, suggesting that microglia may phagocytose synapses even during the wake period but at lower rates. Administration of dexamethasone, a synthetic glucocorticoid, reduces microglial activity, size, and protein expression at ZT0, similar to the control brain at ZT12, and increases synaptic protein levels in the prefrontal cortex, while reducing sleep duration. Along the same lines, noradrenaline (NA) suppresses glutamate-induced phagocytosis in cultured primary microglia, regulating microglial activity, and synaptic elimination. Depleting NA levels with reserpine in vivo increases microglial markers (e.g. complement-3 [C3] and milk fat globule-EGF factor 8 [MFG-E8]), reduces synapsin I in the prefrontal cortex, and increases sleep duration at ZT0, effects that are reversible by restoring NA levels. NA likely influences microglial activity through its effects on the expression of interferon regulatory factor 1 (IRF1), a pro-inflammatory transcription factor, as the knockdown of IRF1 mimicked the effects of NA, linking circadian fluctuations in glucocorticoids and NA to the elimination of weaker synapses during sleep [102]. More recently, the same authors showed that the deficiency of myeloid differentiation factor 88 (MyD88), an adaptor protein transducing signals from Toll-like receptors and IL-1 receptors, reduced the phagocytic activity of microglia. MyD88-deficient mice displayed reduced NREM sleep duration promoting insomnia and depressive-like behaviors [103]. Collectively, these studies suggest that microglia eliminates weaker synapses throughout sleep and, to a less extent, may even do so during the day, supporting the idea that synaptic elimination predominantly occurs during sleep and is facilitated by neurochemical and molecular signals, such as those signaled from microglia [101].

The circadian gene REV-ERBα regulates complement expression and microglial synaptic phagocytosis in the CA3 region of the hippocampus, thereby providing an additional link between circadian regulation and synaptic pruning [104]. The diurnal variation in microglial synaptic phagocytosis is antiphase to REV-ERBα expression, and global deletion of REV-ERBα leads to elevated synaptic phagocytosis [104].

Collectively, evidence indicates that microglial function at synapses is influenced by neuronal activity and vigilance states, and it contributes to the remodeling of neuronal processes. Finally, circadian genes appear to regulate the timing of synaptic phagocytosis, an important process for synaptic plasticity in distinct brain regions.

### Metabolism

The relationship between sleep, metabolism, and glial cell activity has garnered significant scientific interest. These non-neuronal cells are increasingly recognized for their link to circadian physiology via several metabolic pathways, including the insulin signaling and mTOR pathways, the suppression of which has been shown to extend lifespan and healthspan [12, 105–107].

During sleep, metabolic activity is generally reduced as compared to wakefulness, allowing the organism to save and restore energy by increasing protein synthesis and neural energy substrates. On the other hand, metabolic changes can alter sleep amount and sleep architecture, presumably serving to signal sleep needs when metabolic restoration is required. Thus, there is a reciprocal relationship between sleep and metabolism [108, 109].

#### Astrocytes

In mammals, hypothalamic astrocytes can sense nutrients and hormones, integrate metabolic information, and modulate neuronal responses. Hypothalamic astrocytes in mammals regulate energy balance in a circadian manner through distinct pathways [41, 110]. Knockdown of the clock gene *Bmal1* in astrocytes caused alterations in energy balance and glucose homeostasis, and reduced lifespan together with age-dependent astrogliosis, apoptosis of hypothalamic astrocytes, and increased cerebrospinal fluid glutamate and GABA levels in the hypothalamus. Treatment with a GABA_A_-receptor antagonist reduced glutamate levels, delayed the reactive gliosis, as well as the metabolic phenotypes, and expanded the lifespan of the mutants [41]. In a different study, knockdown of *Bmal1* in astrocytes led to sex-specific changes in energy homeostasis and obesity [111]. Under a standard diet, loss of *Bmal1* resulted in a negative energy balance in females, characterized by increased energy expenditure, and elevated brown adipose tissue thermogenesis, hepatic, and white adipose tissue lipogenesis, without affecting circadian locomotor activity, whereas male mutants did not show significant alterations in basal diurnal metabolic rate but exhibited weight gain. However, in female mice on a high-fat diet, the loss of astrocytic *Bmal1* led to a “male-like” obesity phenotype, likely due to reduced energy expenditure and activity, suggesting that astrocytic *Bmal1* regulates metabolic outputs, energy balance, and physiological rhythms in a sex and diet-specific manner [111].

Astrocytes have also been implicated in circadian temperature regulation and thermogenesis, though the main mechanisms still require further investigation. The role of SCN neurons and astrocytes in circadian temperature regulation was studied in the context of the cold-response protein RNA-Binding Motif 3 (Rbm3), which strengthens the circadian amplitude of core clock genes under temperature-entrained conditions. Using ex vivo SCN slices with *Bmal1* deletion, the study showed that *Rbm3-Luc* circadian oscillations are influenced by temperature changes, lengthening at 32 °C and shortening upon return to 37 °C. In contrast, the core clock gene *Per2* remained stable across temperature transitions, indicating the resilience of clock genes to temperature variations and that the transcription of *Rbm3*, controlled by the transcription-translation feedback loop (TTFL), can be predictably modulated by temperature. *Rbm3-Luc* responses require Bmal1 expression, as reduced Bmal1 expression in either neurons or astrocytes abolished Rbm3’s temperature-evoked responses and disrupted circadian rhythmicity; thus, both cell types are necessary for integrating circadian function with temperature, with Bmal1 as a key integrator [112]. Although the mechanisms for glial regulation of thermogenesis and body temperature regulation are not well explored, astrocytic insulin signaling is a potential pathway [113]. This was supported by analysis of mice with an astrocyte-specific insulin receptor knockout, which exhibited reduced energy expenditure and body temperature, underscoring the importance of astrocytic insulin signaling in thermogenesis [113]. Further studies exploring how glial cells contribute to circadian temperature regulation and to determine the specific role of each subtype are warranted.

Recent research has revealed intriguing sex-specific effects of astrocytic insulin signaling on circadian rhythms and energy homeostasis. Male mice lacking insulin receptor (*IRcKO*) maintained normal circadian rhythms but showed reduced nocturnal activity, while female mice exhibited decreased dark phase activity, a prolonged free-running period, and reduced rhythm amplitude [110]. Both sexes displayed food anticipatory activity (FAA) under restricted feeding conditions, similarly to controls, but female *IRcKO* mice showed increased FAA, along with reduced food intake and weight gain, suggesting a sex-specific impairment in metabolic adaptation. Moreover, male *IRcKO* mice had reduced dopaminergic signaling in the striatum, which likely accounted for impaired insulin-induced shifts in FAA as phase shifts could be rescued with a dopamine receptor 2 agonist. In females, this agonist enhanced nocturnal activity and normalized locomotor rhythms [110]. These findings underscore the crucial role of astrocytic insulin signaling in regulating feeding behavior and metabolic adaptation, highlighting significant sex-specific differences in its impact.

### Other glial cell types in *Drosophila* and mammals

In *Drosophila,* a role for glia in energy homeostasis is regulated by *Gart* polypeptide, which is under the control of the CLOCK/CYCLE circadian heterodimer [114]. It coordinates energy intake, storage, and expenditure, thereby impacting the survival of flies under adverse conditions. Downregulation of *Gart* in glial cells led to decreased energy storage, lower survival rates, and decreased survival time under starvation conditions, while the re-expression of this polypeptide effectively improved survival rates and average survival time under starvation stress, indicating the essential role of glia in regulating metabolic processes and stress responses in *Drosophila* [114].

The surface glia of flies, which comprise the BBB, display circadian, and sleep regulation of function in terms of their permeability to proteins and lipids and their metabolic functions [45, 50, 115]. Downregulation of *Pallidin* and other members of BLOC1, which are involved in proteostasis, in BBB glia reduces and delays nighttime sleep. This occurs in a circadian-clock-dependent manner, via mTOR amino acid signaling. Providing essential amino acids by supplementing with leucine-rich food normalized the sleep/wake phenotypes. *Pallidin* in surface glia is also required for GABAergic neuronal activity, connecting essential amino acid availability, and GABAergic regulation of sleep/wake [115].

Efflux transporters, which pump out small lipophilic molecules, in the BBB are under circadian control such that their activity is highest during the animal’s wake hours [44, 46, 116]. This is true in *Drosophila* and mammals and it restricts brain entry of lipophilic molecules to the sleep phase, perhaps serving to protect the brain from xenobiotics that the animal may be exposed to during waking hours [44, 46, 116]. The lipid-soluble steroid hormones ecdysone also crosses the BBB during nighttime and promotes sleep by stimulating lipid metabolism in cortex glia [51]. It is unclear though if the entry of ecdysone is regulated by efflux transporters.

Endocytosis through the *Drosophila* BBB also occurs largely during sleep and appears to be driven by homeostatic mechanisms such that it is promoted by prior wakefulness [44]. The nature of the molecules trafficked is largely lipids, particularly acylcarnitines, which are fatty acids linked to carnitine for mitochondrial transport; these build up in fly heads when endocytosis is blocked. Blocking endocytosis increases sleep and sleep needs, perhaps because more sleep is now required for the clearance of metabolites. Knockdown of lipid transporters *LRP1*&2 or of carnitine transporters *ORCT1*&2 also increases sleep and raises acylcarnitine levels, suggesting that these transporters are impacted by the block in endocytosis [50].

Membrane lipids, including sphingolipids, regulate neurite remodeling in a circadian-dependent manner, facilitating adaptive structural plasticity [48]. In *Drosophila*, glycosphingolipids are degraded by Glucocerebrosidase (GBA), encoded by the *Gba1b* gene. Knockdown of *Gba1b* promotes the accumulation of lysosome protein Ubiquitin, driving lysosomal dysfunction, particularly in glia in the inner optic lobe chiasm and the Mushroom Body neural cortex [48]. This phenotype is specific to glial cells, showing that glia are crucial for glycosphingolipid catabolism. Restoring *Gba1b* expression in ensheathing and perineural glia, but not in astrocytes, mitigated the lysosomal and proteostatic alterations observed in the knockdowns. Additionally, Ubiquitin aggregation follows daily rhythms regulated by circadian clocks, and disruption of these rhythms in *Per* and *Gba1* mutants promotes Ubiquitin accumulation, impairing proteostasis. *Gba1* mutants also exhibit altered lipid profiles, reduced circadian fluctuations, and shorter sleep bouts. Knockout of *Gba1* in glia, as well as specifically removing *Gba1b* in both ensheathing and perineural glia [48], resulted in sleep loss, whereas individual ensheathing and perineural drivers had only a modest effect. This is consistent with the role of barrier glia (perineurial glia are part of the BBB) in regulating sleep [44, 117]. Rescue of sleep deficits was achieved by expressing *Gba1b* in both ensheathing and perineural glia. Thus, these glial subtypes balance lipid trafficking and clearance across the *Drosophila* brain, which is essential for healthy synaptic plasticity [48].

Fatty acid-binding proteins are lipid chaperones that mediate the intracellular dynamics of hydrophobic molecules, playing roles in cell proliferation and survival, inflammation, and metabolism [118]. In mammals, glial *Fabp7* mRNA expression is clock-controlled by *Rev-Erbα* in the hippocampus and the hypothalamus, and by *Bmal1* in the neocortex and hippocampus [119–121]. In a recent review, Gerstner et al. proposed that FABP7 integrates circadian timing of sleep with sleep needs through changes in neuronal-glial interactions [122], likely involving metabolic processes. They showed too that the missense mutation FABP7.T61M is associated with fragmented sleep in humans, a phenotype that is conserved in *Fabp7* KO mice and *Drosophila* that overexpress the human FABP7.T61M mutation specifically in astrocytes, suggesting a conserved molecular pathway involving lipid signaling within astrocytes that regulates sleep across different species [42]. In a different study involving *Drosophila*, manipulation of the conserved FABP ortholog, *dFabp*, through knockdown or overexpression in the developing brain, wing, and eye, impacted survival and cell proliferation and increased apoptosis [43]. Specifically, the knockdown of *dFabp* in glial cells or neurons impairs neuronal development by reducing the thickness of the mushroom body, a center for learning and memory. Furthermore, both knockdown and overexpression of *dFabp* in neurons disrupted gliogenesis. Additionally, while overexpression of *dFabp* in glia supported normal circadian rhythmicity, knockdown in either glia or neurons reduced locomotor activity and increased the percentage of arrhythmic flies compared to controls [43], suggesting that dFabp mediates neuronal development and function, including neuron-glia interactions, to control behavior. In general, studies of the circadian role of FABP7 and related lipid signaling pathways and how they impact metabolic and inflammatory pathways could reveal connections between clock-regulated mechanisms, fatty-acid pathways, and disease, given that *Fabp7* overexpression is linked to various diseases, including sleep and psychiatric disorders [42, 123, 124].

Under stress conditions, neurons transfer fatty acids to glial cells for temporary storage in lipid droplets and catabolism in the mitochondria, promoting detoxification and energy regulation [125, 126]. Recently, a mechanism involving mitochondrial signaling and lipid metabolism in neurons and glia was linked to daily sleep [53]. It was found that at the end of a day of wake, mitochondrial oxidation occurs in glia and not neurons, even though neurons are metabolically active during wake. This is because neurons transfer their oxidative damage to glia, where it accumulates as lipid droplets during sleep; thus, the stress-dependent or pathological scenario mentioned above has a physiological function in that it occurs in a daily sleep:wake cycle. Knocking down fatty acid transport proteins, such as GLaz in glia, reduced the accumulation of lipid droplets in glia and enhanced neuronal mitochondrial oxidation; concomitantly, sleep was reduced and fragmented. Knocking down *Drp1* or other mitochondrial damage control genes in either neurons or glia also disrupted sleep patterns. Mitophagy in both neurons and glia was found to occur in the morning in a sleep-dependent fashion, highlighting the role of sleep in lipid metabolism and mitochondrial turnover across cell types [53]. Given that the fatty acid transport proteins GLaz and NLaz (expressed primarily in neurons) share homology with mammalian apolipoprotein E (*ApoE*), a gene associated with AD [127], deficits in lipid transport from neurons to glia constitute a potential mechanism through which chronic sleep disruption could increase dementia risk in AD patients. These findings emphasize the role of sleep in maintaining brain health and highlight the link between sleep disruption and neurological disorders such as AD.

Autophagy, a cellular self-degradative process that removes misfolded or aggregated proteins and clears damaged organelles, is also regulated by circadian influences and sleep [49, 128, 129]. In *Drosophila*, autophagy regulated by glia modulates circadian structural changes in processes of the pacemaker neurons, highlighting its importance for healthy neuron-glia crosstalk [130]. Autophagy is also regulated by sleep in *Drosophila* although thus far this has only been reported in neurons [129]. In mice, autophagy was shown to be stimulated via BMAL1-dependent mechanisms in astrocytes [49]. Regulation of proteostasis to remove misfolded proteins in different cell types in the brain is critical for healthy brain aging. Thus, understanding the role of circadian and sleep regulation in the control of degradation pathways and their connection to neurodegenerative diseases, which are characterized by the accumulation of misfolded/toxic proteins, is fundamental. In mammals, transcriptomic analysis of *Bmal1* KO mice within Gfap-expressing astrocytes in the ventromedial hypothalamus (VMH) revealed that a high-fat diet triggers cellular stress responses, inducing several proteostasis pathways, including the endoplasmic reticulum-stress pathway, the unfolded protein response, and autophagy, via key transcription factors, *Xbp1* and *Atf1* [131]. This suggests that the astrocytic circadian clock modulates energy balance in the VMH by regulating cellular stress responses.

### Immune system

Sleep is important for recovering from acute stress or illness, enhancing recovery and survival via cellular adaptations [132–135]. This is even true in invertebrates, where the mechanisms underlying this association have been more extensively explored [136–141]. Multiple triggers can induce cellular stress, including sleep loss/disruption and environmental stressors such as extremes of temperature, exposure to ultraviolet (UV) radiation, toxins, pathogenic infections, and mechanical damage, all of which initiate a cascade of molecular changes [142]. Glial cells are pivotal in neuroimmune responses, with increasing evidence linking sleep and glial immune responses [143–145].

In flies, one of the classical immune pathways involves the master immune regulator nuclear factor binding the κ light chain in B-cells (NF-κB), which is a key component of both the Toll and the Imd pathways, crucial for the induction of antimicrobial peptides (AMPs) [146]. Alterations in sleep following injury or infection in *Drosophila* are dependent on NF-κB Relish [137]. How the different glia subtypes in *Drosophila* contribute to NF-κB function is still largely unknown. Only recently, was this question somewhat addressed in a model of traumatic brain injury (TBI) in *Drosophila*; in this model, the injury up-regulates AMP genes that are regulated via Toll, Imd, and JAK–STAT pathways, predominantly in glial cells in wild type flies. In wild-type flies, TBI causes apoptosis, lethality, impaired locomotion, and drives sleep loss and fragmented sleep in a dose-dependent manner (either 1, 5, or 10 strikes to the head). RNAi-mediated knockdown of most AMP classes increases TBI-induced mortality, except for flies lacking Defensin, which survive longer. Additionally, the NF-κB pathway plays a crucial role after TBI, as NF-κB mutants show increased lethality following TBI, but, while they reduce sleep, their sleep and motor functions are similar to those of sham-treated controls, suggesting that the immune response driven by NF-κB aids survival and contributes to TBI-induced sleep and motor deficits [147]. Together, these results indicate that the innate immune response to TBI in *Drosophila* can have both beneficial and detrimental effects. Moreover, the identification of 512 differentially expressed glial genes in the TBI model implicates proteolysis and protein folding pathways, highlighting the essential role of glial proteostasis in the neuroimmune response to TBI [147].

In mammals, microglia are key players in the response to inflammatory challenges, with mounting evidence suggesting that these responses are sleep-mediated [81, 148]. For instance, following inflammatory challenges, mice depleted of microglia sleep more compared to controls [81]. Pharmacological depletion of microglia using PLX5622 initially increases sleep, which normalizes by the second week. Subsequent reintroduction of microglia in mice caused no changes in sleep until a second inflammation-inducing injection [81]. Similar sleep–wake alterations are evident post-intracerebral hemorrhage, where activated microglia induce inflammation and cytokine signaling, impacting sleep patterns [148].

Regarding the molecular pathways that mediate sleep-dependent inflammatory responses, the NOD-like receptor pyrin domain containing 3 (NLRP3) inflammasome pathways seems pivotal. In rats with chronic pain, chronic sleep deprivation aggravated pain behaviors by upregulating the expression of Toll-like receptor 4, NLRP3, and interleukin-1β (IL-1β), thereby activating microglia [149]. This microglial activation and polarization through the NF-κB and NLRP3 inflammasome pathways was shown to be clock regulated by Rev-erbα in BV2 cell experiments [150]. In mice, sleep deprivation increased pain sensitivity and exacerbated post-surgical pain through reactive oxygen species (ROS) elevation, microglial activation, and DNA damage in the spinal cord. ROS inhibition with phenyl-*N*-tert-butylnitrone (PBN) alleviated sleep deprivation-induced hyperalgesia by suppressing microglia activation and NLRP3 inflammasome activity [151]. These findings highlight NLRP3 inflammasome involvement in sleep-induced inflammation and oxidative stress, suggesting that targeting this pathway could provide therapeutic strategies for mitigating inflammation and pain exacerbated by sleep deprivation.

An interesting response of microglia was shown to occur prior to the invasion of the CNS by the intraperitoneally-injected parasite *Trypanosoma brucei*, which causes sickness-sleep behavior, in transgenic mice expressing the enhanced green fluorescent protein under the Cx3cr1 promoter. Microglia respond to this peripheral inflammation by altering their volume and morphology, as evidenced by the increase in somata size and the reduced length of microglial processes. In synchrony with the microglial activation, initial signs of inflammation can be visually observed near the meninges and parenchyma of the neocortex; the meninges become thicker in this region, indicative of meningeal space swelling [152]. Similar anticipatory responses of microglia to a different pathogen, the *Influenza A* virus, have been observed, suggesting that these cells are primed to protect neurons from pathogens entering the brain [153].

Besides sleep-dependent inflammatory responses of microglia, several papers have demonstrated a role for microglial clocks in regulating inflammatory state/rhythms [154–157]. Hippocampal microglia isolated from adult rats possess intrinsic clocks that oscillate throughout the day and can be entrained by glucocorticoids [154, 155]. Microglia clocks rhythmically express inflammatory cytokines, such as TNFα, IL1β, and IL6 mRNA, with peak expression occurring in the middle of the light phase [154]. Additionally, microglia isolated during the light phase are more reactive to immune stimulation [154], suggesting that the endogenous timekeeping mechanisms of microglia regulate neuroinflammatory responses.

As noted above, the clock gene Rev-erbα is implicated in microglial activation and associated inflammatory responses. Primary hippocampal microglia from *Rev-erbα* KO mice showed increased expression of pro-inflammatory transcripts, secondary astrogliosis, and NFκB–related gene expression [157]. In addition, conditioned media from *Rev-erbα*–deficient primary glial cultures negatively impacted neuronal health, causing exacerbated oxidative damage in cultured neurons. *Rev-erbα* KO mice also show altered cortical resting-state functional connectivity, resembling patterns seen in neurodegenerative models [157]. Pharmacological depletion of microglia (using the CSF1R inhibitor, PLX5622) in adult *Rev-erbα* KO mice alleviates hyperactivity, memory impairments, and anxiety/risky-like behaviors, while reducing pro-inflammatory cytokine expression (IL-1β and IL-6). Microglia depletion in adult *Rev-erbα* KO mice reduced microglial branching and decreased CD68 production without affecting astrogliosis in both sexes, with stronger phenotypic and neuroimmune alterations in male mice than in females [156].

Age may cause disruption of diurnal rhythms in microglia and neuroinflammatory processes, as evidenced by altered Per1, Per2, TNFα, and IL1β expression in isolated hippocampal microglia from old rats compared to younger ones [155]. Additionally, aged rats show a loss of diurnal regulation of microglial activation markers (e.g. Iba1 and CD68) and display altered inflammatory responsiveness, characterized by increased expression levels of Iba1, CD68, GFAP, MHCII, and CD200R and reduced CX3CL1 in the aged hippocampus. Furthermore, aged rats show amplified *E. coli*-induced hippocampal IL-1β protein, particularly during the dark phase [155].

In sum, glial immune responses seem to be regulated by both sleep homeostatic mechanisms and circadian rhythms, which involve glial clocks. Growing evidence links glial clock genes to the pathophysiology of neurological diseases, such as Alzheimer’s disease [158–160], tauopathies [161], mood-related behavior [162], and demyelinating disorders like multiple sclerosis [163]. These associations between glial clocks and pathophysiology may have broad mechanistic and therapeutic implications for brain disorders that can include sleep phenotypes such as chronic insomnia [164].

## Concluding Remarks and Future Perspectives

**Figure 2.**
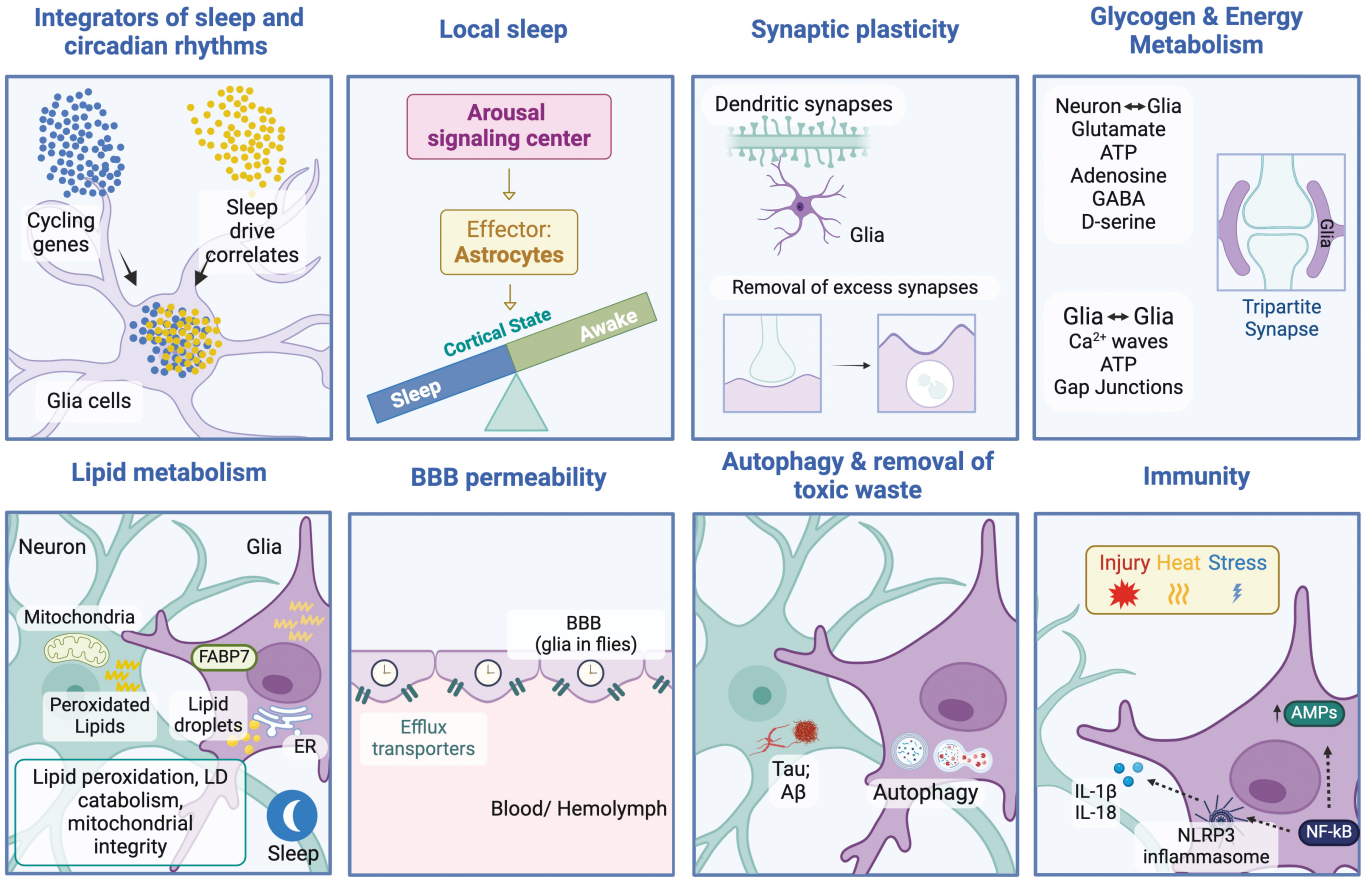
Summary of the diverse circadian and sleep-regulated functions served by glial cells. Glial cells, integrate sleep and circadian rhythms by interacting with neural networks that maintain homeostasis and regulate local sleep dynamics through astrocytic modulation of cortical states. They contribute to synaptic plasticity by removing excess dendritic synapses and support glycogen and energy metabolism through the tripartite synapse, which involves neurotransmitters and signaling molecules such as glutamate, ATP, adenosine, GABA, and D-serine. During sleep, glial cells influence blood–brain barrier permeability, facilitate lipid metabolism for mitochondrial energy production, and enhance autophagic activity to clear toxic waste, such as clearance of Tau and Amyloid-β, thereby playing a neuroprotective role. Additionally, they modulate immune responses by activating pathways such as the NLRP3 inflammasome and NF-kB signaling, underscoring their importance in brain health and homeostasis. Adapted from [45, 46, 54, 122, 165, 166].

Emerging research highlights the pivotal role of glial cells in integrating sleep homeostasis and circadian rhythms, impacting behavior, plasticity, and cognition. Traditionally studied separately, circadian and sleep influences are clearly interconnected, with astrocytes influencing local sleep circuits, and glial cells in general regulating synaptic plasticity, metabolism, temperature, and immune challenges in a clock-dependent manner (Figure 2). Indeed, genetic manipulation studies have established circadian regulation of glial activity, although the mechanisms remain largely unexplored and likely vary from process to process. As many glial processes are also under homeostatic control, we speculate that their circadian timing optimizes their contribution to sleep function in a daily cycle. Notably, sex differences in the circadian and sleep functions of glia should be investigated as hormonal patterns likely have substantial impact on circadian/sleep-regulated metabolic activity in glial cells.

A key area for future research is the role of different glial subtypes. Despite significant research on glial contributions to sleep and circadian rhythms over the past two decades, astrocytes remain the most studied subtype, while the roles of other glial subtypes, such as oligodendrocytes and NG2-glia, in sleep and circadian rhythms are less understood. Additionally, while microglial involvement in sleep regulation and sleep-regulated functions such as immune responses are well-documented, relatively little is known about the circadian outputs of microglia. Likewise, in *Drosophila*, the role of circadian regulation in different glial subtypes remains underexplored. A clock in the BBB glia regulates the activity of efflux transporters, which confers a rhythm of permeability onto small lipophilic molecules, but mostly *Drosophila* glial subtypes have been studied more in the context of sleep.

Another interesting question is whether and how glia modulate neuronal clocks, to shape the timing and amplitude of circadian rhythms and contribute to circadian behaviors. For instance, studying the interaction between glial and neuronal clocks may reveal insights into the synchronization of circadian rhythms throughout the brain. Neuron-glia interactions are likely also relevant for the effects of circadian control and sleep on synaptic plasticity and memory consolidation. And these interactions may, in turn, be regulated by time of day or behavioral state, and even by age. Age-related changes could contribute to sleep disturbances in elderly populations.

Understanding the translatability of these mechanisms in human contexts is crucial, as it may lead to therapies for mitigating neural damage from sleep disruptions, a prevalent issue in current society. For instance, investigating glial involvement in sleep-related neuroinflammation and neurodegeneration could lead to early diagnostic markers or treatment strategies. Continued exploration in this field promises significant insights into the complex interplay of glial cells, sleep, and circadian biology.

## Data Availability

No new data were generated or analysed in support of this research.
